# Membranous expression of Her3 is associated with a decreased survival in head and neck squamous cell carcinoma

**DOI:** 10.1186/1479-5876-9-126

**Published:** 2011-07-29

**Authors:** Mikiko Takikita, Ran Xie, Joon-Yong Chung, Hanbyoul Cho, Kris Ylaya, Seung-Mo Hong, Christopher A Moskaluk, Stephen M Hewitt

**Affiliations:** 1 Tissue Array Research Program, Laboratory of Pathology, National Cancer Institute, National Institutes of Health, Bethesda, MD 20892, USA; 2 Applied Molecular Pathology Laboratory, Laboratory of Pathology, National Cancer Institute, National Institutes of Health, Bethesda, MD 20892, USA; 3 Department of Pathology, Johns Hopkins Medical Institutions, Baltimore, MD 21287, USA; 4 Department of Pathology, University of Virginia Health System, Charlottesville, VA 22908, USA

## Abstract

**Background:**

Head and neck squamous cell carcinoma (HNSCC) still remains a lethal malignancy benefiting from the identification of the new target for early detection and/or development of new therapeutic regimens based on a better understanding of the biological mechanism for treatment. The overexpression of Her2 and Her3 receptors have been identified in various solid tumors, but its prognostic relevance in HNSCC remains controversial.

**Methods:**

Three hundred eighty-seven primary HNSCCs, 20 matching metasis and 17 recurrent HNSCCs were arrayed into tissue microarrays. The relationships between Her2 and Her3 protein expression and clinicopathological parameters/survival of HNSCC patients were analyzed with immunohistochemistry.

**Results:**

Her3 is detected as either a cytoplasmic or a membranous dominant expression pattern whereas Her2 expression showed uniform membranous form. In primary tumor tissues, high membranous Her2 expression level was found in 104 (26.9%) cases while positive membranous and cytoplasmic Her3 expression was observed in 34 (8.8%) and 300 (77.5%) samples, respectively. Membranous Her2 expression was significantly associated with histological grade (*P *= 0.021), as grade 2 tumors showed the highest positive expression. Membranous Her3 over-expression was significantly prevalent in metastatic tissues compared to primary tumors (*P *= 0.003). Survival analysis indicates that membranous Her3 expression is significantly associated with worse overall survival (*P *= 0.027) and is an independent prognostic factor in multivariate analysis (hazard ratio, 1.51; 95% confidence interval, 1.01-2.23; *P *= 0.040).

**Conclusions:**

These results suggest that membranous Her3 expression is strongly associated with poor prognosis of patients with HNSCC and is a potential candidate molecule for targeted therapy.

## Background

The majority of tumors that arise in the head and neck region are squamous cell carcinomas arising from the upper aerodigestive tract epithelium. Progressive local spread of head and neck squamous cell carcinoma (HNSCC) affects the highly critical functions of speech, swallowing and respiration. HNSCC has a 50% disease specific mortality in the USA[1], claiming 11,000 lives a year, and also represents one of the top ten cancers worldwide[2]. Despite significant advances in the medical and surgical treatment of these cancers, this statistic has remained stable for decades. Novel, more effective therapeutic strategies to improve overall survival are urgently needed.

Recently, the use of targeted agents against molecular markers belonging to the human epidermal growth factor receptor (HER) family has been integrated into the treatment of protocols for many malignancies. HER family consists of four homologue members (EGFR/Her1/erbB1, Her2/erbB2, Her3/erbB3, and Her4/erbB4). All share a common structure, with an extracellular ligand-binding domain, a transmembrane domain, and an intracytoplasmic tyrosine kinase domain[3-5]. Ligand binding to these receptors induces the formation of receptor homodimers and heterodimers, and thereby activates numerous downstream pathways regulating diverse processes including differentiation, migration, proliferation, and survival.

Her2 has an extracellular domain, but appears to lack ligand-binding activity, while Her3 has a non-functional kinase domain and has no catalytic activity. Her2-Her3 function by formation of a heterodimeric complex which actives an oncogenic signaling pathway (e.g. PI3/AKT pathway)[6]. Even in its over-expressed and oncogenic state Her2 does not escape its dependency on HER family partners, and Her3 plays an important and necessary function in Her2-mediated tumorigenesis[7]. As the HER pathway contributes significantly to progression of cancers, its family members serve as a group of anti-cancer drug target with great clinical potential. Current therapeutic efforts against the HER family are focused on small molecule tyrosine kinase inhibitors (TKIs) and humanized or chimeric monoclonal antibodies (mAbs)[8,9]. Recent studies revealed that Her3 is the principle mediator of TKI resistance. TKIs effectively prevent auto-phosphorylation of EGFR and Her2 in tumor cells, however, the transphosphorylation of Her3 is only transiently suppressed and Her3 ultimately escapes inhibition by TKIs in Her2 over-expressing tumor cells[10]. Consequently, the Her3 resistance causes PI3/Akt pathway resistance, tumor survival, and escape from proapoptotic consequences of the loss of oncogenic Her2 signaling.

Her2 over-expression in HNSCC has been reported, [11] but there are few studies on Her3 expression in HNSCC[12]. Clinical studies with agents targeting HER proteins have been performed in patients with HNSCC, with promising results[13-15]. However, the prognostic significance and the potential as biomarkers of Her2 and Her3 in HNSCC remains undetermined. In the present study, protein expression levels of Her2 and Her3 were interrogated on a tissue microarray (TMA) of surgically removed samples of HNSCC by immunohistochemistry (IHC). The relationships between protein expression and clinicopathological parameters/survival of HNSCC patients were also analyzed.

## Materials and methods

### Patients and tumor samples

A total of four hundred twenty four formalin fixed and paraffin embedded tumor specimens with HNSCC were obtained from the archives of the Pathology Department of the University of Virginia Health System and were assembled into TMA blocks containing: 387 primary HNSCC tissues, 20 matching metastatic tissues and 17 recurrent HNSCC tissues. The clinical information of these patients was obtained from the University of Virginia Cancer Registry. Material was obtained with appropriate human protection approvals from the institutional review board of University of Virginia Health System and office of Human Subjects Research at the NIH. Information on post-operative radiation and/or chemotherapy, and performance status of patients was unavailable for analysis.

### Tissue microarray construction

TMAs were constructed from archival formalin fixed, paraffin embedded tissue blocks. For each tumor, a representative tumor area was carefully selected from a hematoxylin and eosin stained section of a donor block which as previously described[16]. Four 0.6 mm diameter cores were retrieved from selected regions of donor blocks from each case and transplanted to the recipient block using a manual tissue arrayer (Beecher Instruments, Silver Spring, MD). Multiple 5-μm thick sections were cut with a microtome and H&E staining of TMA slides were examined every 50^th ^sections for the presence of tumor cells.

### Western blot analysis of Her3 antibody

For three cell lines, A549, MCF7 and BxPC3, a total 4 × 10^7 ^cells were rinsed twice with ice-cold PBS and added 0.5 ml of the Protein Extraction Solution RIPA (Pierce Biotechnology, Rockford, IL). After incubation for 30 min on ice, cells were scraped and centrifuged. Protein concentrations were measured by the BCA protein assay kit (Pierce Biotechnology). To determine the specificity of anti-Her3 antibody, 30 μg of protein were separated by 4-12% NuPAGE^®^Novex Bis-Tris polyacrylamide gel electrophoresis and transferred to nitrocellulose membrane (Invitrogen, Carlsbad, CA). The membranes were blocked with 5% nonfat dry milk in TBST (50 mM Tris, pH 7.5, 150 mM NaCl, 0.05% Tween-20) for 1 h, washed, and subsequently incubated overnight at 4°C in TBST with 5% BSA containing anti-Her3 antibodies (RTJ.2, mouse monoclonal; Santa Cruz Biotechnology, Santa Cruz, CA; dilution 1:1000). Her3 expressional signals were detected with horseradish peroxidase-labeled anti-mouse antibodies (Chemicon International) and enhanced with SuperSignal Chemiluminescence kit (Pierce Biotechnology).

### Immunohistochemistry and scoring

To investigate the significance of Her2 and Her3 expression in HNSCC, 4-micron histologic sections of the TMAs were stained by IHC. Briefly, tissue sections were deparaffinized and hydrated in xylene and serial alcohol solutions, respectively. Endogenous peroxidase was blocked by incubation in 3% H_2_O_2 _for 10 min. Antigen retrieval was performed in a steam pressure cooker with prewarmed antigen retrieval buffer pH 6 (DakoCytomation, Carpinteria, CA) at 95°C, for 10 min and 40 min, for Her3 and Her2 staining respectively. To minimize non-specific staining, the section was incubated with protein block (DakoCytomation) for 15 min. After washing with TBST, the specimen was incubated with anti-Her3 antibodies (RTJ.2, mouse monoclonal; Santa Cruz Biotechnology, Santa Cruz, CA; dilution 1:500) overnight at 4°C, anti-Her2 antibodies (c-erbB2, A0485, rabbit polyclonal: Dako; dilution 1:750) at room temperature for 30 min. Antigen-antibody reactions were detected with DAKO LSAB^®^+ peroxidase kit (Dako). The stain visualized using 3,3'-diaminobenzidine plus (Dako) and was lightly counterstained with hematoxylin, dehydrated in ethanol, and cleared in xylene. Appropriate negative controls were concurrently performed, and the TMAs included appropriate positive control tissues. The slides were covered and observed under a light microscope (Axioplot, Carl Zeiss, Jena, Germany). Her3 assessment included manual qualitative interpretation of both membranous and cytoplasmic staining. Her2 (membranous) staining and Her3 membranous staining were scored according to the commonly applied criteria of Her2 membranous staining (0,+1, +2, +3) and further dichotomized as either negative (score 0) or positive (+1, +2, or +3)[17]. For assessment of Her3 cytoplasmic staining, two scores were assigned to each core. (a) the staining intensity [categorized as 0 (absent), 1 (weak), 2 (moderate), or 3 (strong)] and (b) the percentage of positively stained epithelial cells [scored as 0 (0% positive), 1 (1-25%), 2 (26-50%), 3 (51-75%), and 4 (76-100%)]. An overall protein expression score was calculated by multiplying the intensity and positivity scores (overall score range, 0-12). This overall score for each patient was further simplified by dichotomizing it to negative (overall score < = 3) or positive (score of > = 4). Consensus review by two pathologist (MT and SMH) was conducted.

### Statistical analysis

The Chi-square test was applied to test the possible association between the expression of Her2/Her3 and the clinicopathologic parameters. The Mann-Whitney *U*-test was used for the analysis of the relationship between Her2/Her3 expression and the patient's age. Kaplan-Meier curves were plotted to assess the effect of Her2/Her3 expression on overall survival. Different survival curves were compared using the log-rank test. Multivariate proportional Cox models were applied to assess the prognostic significance of Her2, Her3, primary tumor sites, histological grading, gender and age. *P*< 0.05 was regarded as statistically significant. All statistical analyses were performed using the SPSS for Window (16.0) package (SPSS, Chicago, IL).

## Results

### Clinicopathological features of patients

Clinicopathological characteristics of cases are summarized in Table 1. The ages of the patients ranged from 20 to 95 years (mean, 61 years). Two hundred and ninety two patients were men and 94 were women. Eighty nine cases were grade 1 tumors, 230 grade 2 and 59 grade 3. Approximately 90% of the patients were either laryngeal or oral cancers. The majority of metastatic tissues (85.0%) were obtained from lymph nodes showing metastatic spread from primary HNSCC. Information about tumor staging was not available for this study group.

**Table 1:** Characteristics of Patients

Variables	Number (%)
**Primary tumor**	387 (100)
**Age**	
Median	61
Range	20 - 95
**Gender**	
Male	292 (75.5)
Female	95 (24.5)
**Tumor sites**	
Larynx	183 (47.3)
Oral cavity	157 (40.6)
Pharynx	23 (5.9)
Nasal cavity	16 (4.1)
Salivary gland	8 (2.1)
**Histological grading**	
Grade 1	89 (23.5)
Grade 2	230 (60.8)
Grade 3	59 (15.7)
**Lymph node metastasis**	
No	363 (95.8)
Yes	16 (4.2)
**Metastatic tumor**	20 (100)
Lymph node	17 (85.0)
Salivary gland	3 (15.0)
**Recurrent tumor**	17 (100)

### Expression of Her2 and Her3

**Figure 1:**
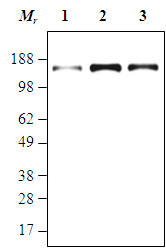
**Characterization of anti-Her3 antibodies by western blotting**. Three cell lines (lung; A549, Breast; MCF7, and Pancreatic; BxPC3) were tested with 30 μg of cell line lysates. 1, A549; 2, MCF7; 3, BxPC.

We performed western blotting in three cell lines (A549, BxPC3 and MCF7) to verify the specificity and capability of the anti-Her3 antibodies. Western blotting experiments showed that of the three cell lines tested, MCF7 cells had high levels of Her3, BxPC3 cells had intermediate Her3, and A549 cells had low Her3 (Figure 1).

**Figure 2:**
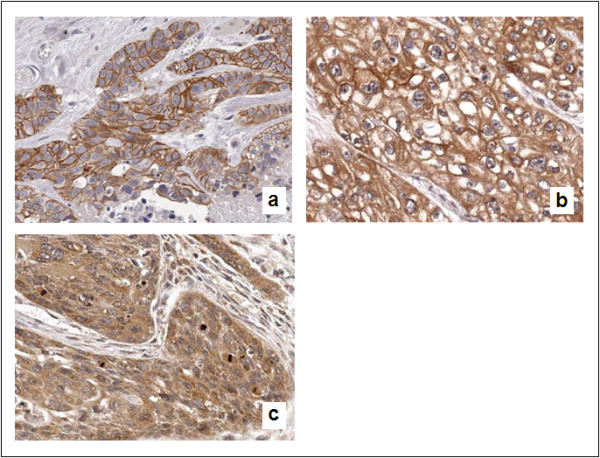
**Representative images of immunohistochemistry for Her2 (*a*, membranous staining and Her3 (*b*, membranous and cytoplasmic staining, and *c*, predominant cytoplasmic staining) 400 × magnification**.

We analyzed the expression pattern of Her2 and Her3 proteins using IHC in 424 tumor samples. Twenty patients were represented by both primary tumors as well as metastatic lesions. Her2 was expressed exclusively in the cell membrane (Figure 2a). Her3 staining was observed in the both cell membrane and cytoplasm (membranous staining; Figure 2b) or predominantly in the cytoplasm (cytoplasmic staining; Figure 2c). Negative control sections demonstrated no staining (data not shown). Her2 staining was scored based on the scoring system applied to breast cancers, with scores of 0,+1, +2, +3 for increasing intensity and "continuity" of staining of the cell membrane[17]. For quantification of Her3 staining, we scored two compartments of the cell - the cell membrane, using the same scoring system as applied for Her2, and the cytoplasm. For the cytoplasmic staining, we scored both the intensity (0 (negative) to 3 (strong)) and the percentage of tumor cells with the dominant intensity staining pattern (0 (none) to 4). These two scores were then multiplied (range 0 to 12). Her2 staining was considered positive for tumors with scores greater than or equal to 1, and Her3 staining was considered positive with tumors with composite scores of greater than or equal to 3.

Her2 positive staining was observed in 104 (26.9%) of primary tumor cases. Her2 expression was more frequently observed in Grade 2 HNSCC tumors (*P *= 0.02). There was no relationship between membranous Her2 protein expression and clinicopathological parameters (Table 2).

**Table 2:** Correlations between Her2/Her3 Expression and Clinicopathological Parameters

	Her2 (membranous staining)			Her3 (membranous staining)			Her3 (cytoplasmic staining)		
	**Negative (%)**	**Positive (%)**	***P***	**Negative (%)**	**Positive (%)**	***P***	**Negative (%)**	**Positive (%)**	***P***

**Primary tumors**	283 (73.1)	104 (26.9)		353 (91.2)	34 (8.8)		87 (22.5)	300 (77.5)	
**Age**									
Median	61.0	61.0	0.866	60.0	63.1	0.140	61	61	0.972
Range	23 - 88	20 - 95		20 - 95	41 - 81		24 - 95	20 - 88	
**Gender**									
Male	217 (74.3)	75 (25.7)	0.355	271 (92.8)	21 (7.2)	0.052	66 (22.3)	227 (77.7)	0.856
Female	66 (69.5)	29 (30.5)		82 (86.3)	13 (13.7)		22 (23.2)	73 (76.8)	
**Primary tumor sites**									
Larynx	137 (74.9)	46 (25.1)	0.557	164 (89.6)	19 (10.4)	0.345	39 (21.3)	144 (78.7)	0.541
Oral cavity	116 (73.9)	41 (26.1)		147 (93.6)	10 (6.4)		36 (22.9)	121 (77.1)	
Pharynx	14 (60.9)	9 (39.1)		21 (91.3)	2 (8.7)		8 (34.8)	15 (65.2)	
Nasal cavity	10 (62.5)	6 (37.5)		13 (81.3)	3 (18.7)		2 (12.5)	14 (87.5)	
Salivary gland	6 (75.0)	2 (25.0)		8 (100)	0 (0.0)		2 (25.0)	6 (75.0)	
**Histological grading^1^**									
Grade 1	71 (79.8)	18 (20.2)	0.021	85 (95.5)	4 (4.5)	0.150	24 (27.0)	65 (73.0)	0.244
Grade 2	157 (68.3)	73 (31.7)		208 (90.4)	22 (9.6)		54 (23.5)	176 (76.5)	
Grade 3	49 (83.1)	10 (16.9)		51 (86.4)	8 (13.6)		9 (15.3)	50 (84.7)	
**Lymph node metastasis^2^**									
No	265 (73.0)	98 (27.0)	0.343	331 (91.2)	32 (8.8)	0.430	82 (22.6)	281 (77.4)	0.295
Yes	13 (81.2)	3 (18.8)		14 (87.5)	2 (12.5)		5 (31.2)	11 (68.8)	

^1^Histological grading data were available for 378 cases (97.7%).

^2^Lymph node metastases data were available for 379 cases (97.9%).

Thirty four (8.8%) of primary tumors samples demonstrated membranous staining for Her3. Her3 was not differentially expressed in primary tumors from different sites, including larynx, oral cavity, pharynx, nasal cavity and salivary gland. Likewise, membranous Her3 expression was not associated with histological grade. In contrast, cytoplasmic Her3 staining was observed in 300 (77.5%) of primary tumor samples. However, there was no association between cytoplasmic Her3 staining and any of the clinicopathological parameters examined (Table 2). When comparing primary tumor samples and matching metastatic samples, significant differences in Her3 membranous staining were observed (Table 3, *P *= 0.003). Neither membranous nor cytoplasmic Her3 expression showed correlation with membranous Her2 staining (Spearman correlation, *P *= 0.068 and *P *= 0.621, respectively).

**Table 3:** IHC Expression of Her2 and He3 in Primary Tumors, Paired Metastatic Carcinomas and Recurrent HNSCC

	Primary (n = 387)	Metastatic (n = 20)	Recurrent (n = 17)	*P *value
**Her2 (membranous)**				
Negative	283 (73.1)	11 (55.0)	10 (58.8)	0.104
Positive	104 (26.9)	9 (45.0)	7 (41.2)	
**Her3 (membranous)**				
Negative	353 (91.2)	14 (70.0)	17 (100)	0.003
Positive	34 (8.8)	6 (30.0)	0 (0)	
**Her3 (cytoplasmic)**				
Negative	87 (22.5)	6 (30.0)	3 (17.6)	0.649
Positive	300 (77.5)	14 (70.0)	14 (82.4)	

### Prognostic significance and Her2 and Her3 expression

**Figure 3:**
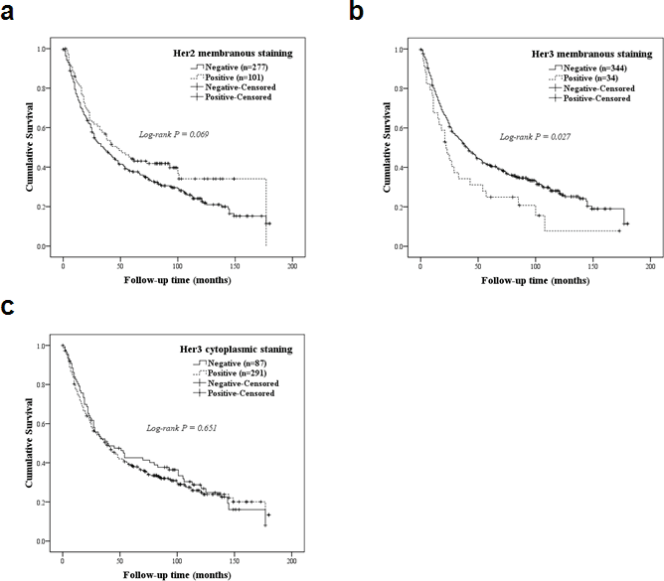
**Kaplan-Meier survival analyses of HNSCC according to Her2 and Her3 expression**. (*a, c*) The Log-rank test did not distinguish the patients with tumors that expressed high levels and low levels of Her2 membranous and Her3 cytoplasmic staining. (*b*) Patients with tumors displaying positive Her3 membranous expression (median survival, 22 months; *n *= 34) had a significantly worse survival time than those with tumors displaying negative membranous Her3 expression (median survival, 40 months; *n *= 344; log-rank test, *p *= 0.027).

Clinicopathological and outcome information was available for 378 (97.7%) of primary HCSCC patients. The length of follow-up time ranged from 1 to 180 months, and median survival at last follow-up was 35 months. Kaplan-Meier survival analyses for patients with different IHC scores are shown in Figure 3. For patients with primary tumors, Her2 staining discriminated survival with borderline significance (Log-rank test, *P *= 0.069), with Her2 negative patients having a worse outcome. In contrast, the patients with positive membranous Her3 expression (median survival, 22 months) had a significantly worse survival time than those with negative membranous Her3 expression (median survival, 40 months; log-rank test, *P *= 0.027). Patients with positive membranous Her3 expression had 1-, 3-, and 5-year survival rates of 66%, 33%, and 24%, respectively, whereas those with negative membranous Her3 expression had 1-, 3-, and 5-year survival rates of 74%, 51%, and 40%, respectively.

The prognostic relevance of Her3 was assessed using a multivariate proportional hazard model adjusted for the clinicopathologic parameters of age, gender, histological grading, primary tumor sites and, lymph node metastasis. Her3 membranous staining positive (hazard ratio, 1.51; 95% confidence interval, 1.01-2.23; *P *= 0.040), age (hazard ratio, 1.02; 95% confidence interval, 1.01-1.03; *P *= 0.001) and primary tumor site were independent prognostic predictors (Table 4). Table 5 shows the results in terms of overall survival and hazard ratio in subsets of patient stratified according to Her2 and Her3 membranous staining status. A total of 262 patients (67.7%) had tumors that were negative for both Her2 and Her3, while co-over-expression of both markers was detected in 13 patients (3.4%). Compared to patients with tumors negative for Her3, patients with tumors positive for Her3 showed a trend toward worse survival irrespective of Her2 staining result. The median survival period was 51.0 months in patients with Her2 positive and Her3 negative cancers, which was statistically significant (*P *= 0.02).

**Table 4:** Prognostic Factors in a Univariate and Multivariate Proportional Hazard Model of The Cox Regression

	Univariate analysis	Multivariate analysis
**Her2 membranous staining**	NS	NS
**Her3 membranous staining**	1.54 (1.04-2.28), 0.027	1.51 (1.01-2.23), 0.040
**Her3 cytoplasmic staining**	NS	NS
**Age**	1.02 (1.01-1.03), 0.001	1.02 (1.01-1.03), 0.001
**Gender**	NS	NS
**Tumor site**		
Larynx vs. Others	NS	NS
Oral cavity vs. Others	NS	NS
Pharynx vs. Others	1.87 (1.17-3.00), 0.008	2.05 (1.28-3.29), 0.003
Nasal cavity vs. Others	0.43 (0.19-0.98), 0.045	0.43 (0.19-0.97), 0.044
Salivary gland vs. Others	NS	NS
**Histological grading**	NS	NS
**Lymph node metastases**	NS	NS

Data are presented as overall survival hazard ratio (95% confidence interval), *P *Value; NS, not significant.

**Table 5:** Outcome of HNSCC Patients According to The Combined Status of Her3 and Her3 Membranous Staining

Tumor markers	Total	Median OS (months)	Hazard Ratio (95% CI)	*P *value
**Her2-/Her3-**	262	36.0	1.13 (0.87-1.47)	0.342
**Her2+/Her3-**	91	51.0	0.70 (0.51-0.95)	0.020
**Her2-/Her3+**	21	25.0	1.53 (0.95-2.47)	0.074
**Her2+/Her3+**	13	22.0	1.48 (0.78-2.79)	0.216

## Discussion

The human epidermal growth factor receptor (EGFR) family of receptor tyrosine kinases, including EGFR, Her2, and Her3, is a potent target for antitumor strategies as it plays a critical role in HNSCC tumor cell growth, survival, invasion, metastasis and angiogenesis. Numerous pharmaceutical approaches have been undertaken to treat various human cancers using drugs that target EGFR family and more than 10 agents are in clinical trials[18-20]. However, current EGFR-targeted therapeutics have had much narrower efficacy than initially predicted based on preclinical models. Due to the limited clinical benefit of current anti-EGFR family therapies, better understanding of EGFR family members is required to develop improved clinical benefit for cancer patients.

Expression of EGFR family members is highly regulated, and outside of the bone marrow, expression is generally low, with increased expression in tumors commonly characterized as over-expression, as it reflects an increase above the baseline expression encountered in the majority of tissues. We attempted to apply a scoring system that reflected the tumor-associated increase in expression of Her2 and Her3, using cut-offs of greater than or equal to +1 (range 0 to +3)for membranous staining of Her2 and Her3, and greater than or equal to 3 (range 0 to 12) for cytoplasmic expression of Her3.

Her2 gene amplification and over-expression has been reported in approximately 30% of breast cancers and in several other tumors, including ovarian, gastric, colorectal cancers[21-24]. In HNSCC, Her2 over-expression has been described previously, although reports on its clinical relevance are less conclusive[11,25-34]. In the present study, we analyzed the expression of Her2 in primary HNSCC and corresponding metastatic tissues by IHC techniques. With a cut-off level between score 0 (negative) and score 1, 2, and 3 (positive) and found Her2 protein expression in 26.9% of primary tumor cases, which is relatively consistent with previous reports[11,30]. However, the frequency of Her2 over-expression decreased from 26.9% to 9.3% if we set cut-off level between score 0 and 1 (negative) and score 2 and 3 (positive), which is conventionally used in breast cancer. The scoring system for Her2 expression in breast cancer does not necessarily translate effectively to other tumor types, and alternative approaches to scoring may be more efficacious[35].

We also found that Her2 expression in our samples was mostly detected in the membrane, and there was lack of cytoplasmic staining. Although Her2 cytoplasmic expression in HNSCC and other cancers has been reported in the previous studies, its interpretation is currently not clear[27,30,36]. In case of breast cancer, membranous staining is the criterion for positivity[17] Another interesting finding is that Her2 expression was associated with histological grade, with the most frequent Her2 expression observed in grade 2 tumors, when cut-off levels are set between score 0 (negative) and score 1, 2, and 3 (positive). However, the association disappears using in HNSCC the conventional cut-off levels for breast cancer, which is consistent with the previous study[11]. These results support the approach that scoring parameters should be carefully considered depending on types of cancers.

The prognostic significance of Her2 expression in HNSCC remains to be elucidated. Some investigators have shown that there was no significant correlation between Her2 over-expression and clinicopathological factors[26,27,34]. The findings of current study are consistent with those previous reports, although conflicting outcomes have been also reported. High frequency of Her2 expression was reported in the patients with HNSCC and it was significantly associated with positive lymph node status and advanced stage[11,28,29]. As there was no correlation between Her2 expressions and most of the clinicophaological parameters, and there was no relation between Her2 over-expression and worse survival of the patients, this suggests that the expression status of Her2 alone might not be a good prognostic predictor in HNSCC.

Another EGFR family, Her3, is one of the most interesting targets for inhibition of EGFR signaling because Her2-Her3 heterodimer constitutes the most active signaling dimer in this family[37]. Nevertheless, the efforts to develop new therapeutic agents that target Her3 in HNSCC or other cancers have lagged behind because of its impaired kinase activity. The dimerization of Her3 with other EGFR family members is required for activating signal pathways. Her2 is regarded as a preferred partner, and requires Her3 to promote cell proliferation. As Her3 and Her2 are mutually dependent proteins and function in complementary manner, but the combination of Her2 and Her3 expressions may be a potentially more useful biomarker in HNSCC than the status of Her2 or Her3 expression alone.

The effect of Her3 expression in HNSCC has been studied previously, [12] but its significance as biomarker had remained undetermined. Several studies showed significant correlation between Her3 over-expression and decreased survival of patients with colorectal, gastric, lung, ovarian and breast cancer,[17,38-40] although conflicting results have been also reported in breast cancer[41-44]. In the present study, Her3 expression was observed predominantly in the cytoplasm (77.5% of primary tumors) and less frequently (8.8%) in the cell membrane of tumor cells. Moreover Her3 over-expression significantly increased in metastatic tissues (30.0%) compared to primary tumors (8.8%). We also found that the Her3 membranous over-expression was significantly correlated with worse survival and was an independent predictive factor in multivariate analysis[45]. Combining both Her2 and Her3 staining result, the patients with Her2 positive and Her3 negative tumors had significantly long survival (*P *= 0.020). We are limited by the lack of information on the staging of primary HNSCC. Tumor staging is important because the stage at diagnosis is the most powerful predictor of survival. However, our findings are,, to our knowledge, the first report of the relationship between both Her2 and Her3 to survival in HNSCC.

The staining pattern of Her3 is not entirely clear, although membranous expression of EGFR and Her2 is regarded as an important parameter. Some investigators have reported predominant cytoplasmic Her3 staining in esophageal,[46] ovarian,[47] whereas cytoplasmic and membranous expression pattern have been reported in colorectal,[48] gastric[40] and breast cancer[43]. In the case of HNSCC, Wei *et al. *reported that Her3 staining was restricted in cytoplasm in laryngeal carcinoma[12]. The discrepancies may be partially explained by the difference of antibodies used for staining or method of assessment for determination of Her3 status. So far, there are no standardized methods of Her3 staining and scoring, our findings suggest the importance of membranous expression of Her3.

## Conclusions

Her3 membranous protein expression was associated with poor prognosis and may represent a new influential parameter on prognosis, independent from the established clinical parameters. In this study, our results showed that Her3 as a potential target for HNSCC therapy development and interference with its function may offer a novel and promising approach to improve clinical patient outcome.

## List of abbreviations used

EGFR: epidermal growth factor receptor; HER: human epidermal growth factor receptor; HNSCC: head and neck squamous cell carcinoma; IHC: immunohistochemistry; TKI: tyrosine kinase inhibitor; TMA: tissue microarray.

## Competing interests

The authors declare that they have no competing interests.

## Authors' contributions

J-YC and SMH conceived of the study and devised the experimental design. SMH and CM designed and build the tissuemicroarrays. MT, J-YC, and YK performed experiments. RX, HC, J-YC and SMH performed data analysis for experiments and clinical records. MT, RX and J-YC drafted the final version of the manuscript and figure legends. SMH revised the figures, added critical content to the discussion and was responsible in revising all portions of the submitted portion of the manuscript. All authors read and approved the final manuscript.
